# Heat-Inactivated *Akkermansia muciniphila* Improves Gut Permeability but Does Not Prevent Development of Non-Alcoholic Steatohepatitis in Diet-Induced Obese Ldlr−/−.Leiden Mice

**DOI:** 10.3390/ijms23042325

**Published:** 2022-02-19

**Authors:** Martine C. Morrison, Eveline Gart, Wim van Duyvenvoorde, Jessica Snabel, Mette Juul Nielsen, Diana Julie Leeming, Aswin Menke, Robert Kleemann

**Affiliations:** 1 Department of Metabolic Health Research, The Netherlands Organisation for Applied Scientific Research (TNO), 2333 CK Leiden, The Netherlands; eveline.gart@tno.nl (E.G.); wim.vanduyvenvoorde@tno.nl (W.v.D.); jessica.snabel@tno.nl (J.S.); aswin.menke@tno.nl (A.M.); robert.kleemann@tno.nl (R.K.); 2 Human and Animal Physiology, Wageningen University, 6708 WD Wageningen, The Netherlands; 3 Nordic Bioscience, Biomarkers and Research, 2730 Herlev, Denmark; mju@nordicbio.com (M.J.N.); djl@nordicbio.com (D.J.L.); 4 Department of Vascular Surgery, Leiden University Medical Center, 2333 ZA Leiden, The Netherlands

**Keywords:** non-alcoholic steatohepatitis, fibrosis, diet-induced, obesity, gut permeability, *Akkermansia muciniphila*, prebiotic, probiotic, microbiota, type IV collagen

## Abstract

The development of non-alcoholic steatohepatitis (NASH) has been associated with alterations in gut microbiota composition and reduced gut barrier function. *Akkermansia muciniphila* is a gut microbe that is thought to have health-promoting properties, including the ability to improve gut barrier function and host metabolism, both when administered live and after heat-inactivation. We questioned whether heat-inactivated *A. muciniphila* may reduce NASH development. Ldlr−/−.Leiden mice, a translational, diet-induced model for NASH, were fed a NASH-inducing high-fat diet (HFD) supplemented with heat-inactivated *A. muciniphila*. After 28 weeks, effects of the treatment on obesity and associated metabolic dysfunction in the gut (microbiota composition and permeability), adipose tissue, and liver were studied relative to an untreated HFD control. Treatment with heat-inactivated *A. muciniphila* did not affect body weight or adiposity and had no effect on plasma lipids, blood glucose, or plasma insulin. Heat-inactivated *A. muciniphila* had some minor effects on mucosal microbiota composition in ileum and colon and improved gut barrier function, as assessed by an in vivo functional gut permeability test. Epidydimal white adipose tissue (WAT) hypertrophy and inflammation were not affected, but heat-inactivated *A. muciniphila* did reduce hypertrophy in the mesenteric WAT which is in close proximity to the intestine. Heat-inactivated *A. muciniphila* did not affect the development of NASH or associated fibrosis in the liver and did not affect circulating bile acids or markers of liver fibrosis, but did reduce PRO-C4, a type IV collagen synthesis marker, which may be associated with gut integrity. In conclusion, despite beneficial effects in the gut and mesenteric adipose tissue, heat-inactivated *A. muciniphila* did not affect the development of NASH and fibrosis in a chronic disease setting that mimics clinically relevant disease stages.

## 1. Introduction

Non-alcoholic fatty liver disease (NAFLD) is an obesity-associated chronic liver disease, with an estimated global prevalence of 25% that is rapidly increasing worldwide [1,2,3]. NAFLD encompasses a spectrum of liver disease, ranging from the relatively benign non-alcoholic fatty liver (NAFL) to non-alcoholic steatohepatitis (NASH), which is characterized by inflammation and fibrosis in addition to fat accumulation and can progress to hepatocellular carcinoma [4].

Both obesity and NAFLD/NASH are associated with alterations in gut microbiota composition [5,6]. The detrimental alteration of gut microbiota composition (dysbiosis) is thought to drive development and progression of NASH [7] through increased permeability of the intestinal epithelial barrier [5], allowing leakage of gut-derived factors into circulation that may have pro-inflammatory effects in the liver [8]. An altered microbiota may also affect the formation of secondary bile acids, some of which are associated with NASH [9]. The abundance of the intestinal bacterium *A. muciniphila*, which is thought to have health-promoting properties [10], is decreased in obesity and associated metabolic diseases [11]. Conversely, treatment with live *A. muciniphila* can attenuate the development of obesity and gut barrier dysfunction in short-term (4 weeks) high-fat diet (HFD)-feeding studies in normolipidemic wildtype mice, along with improvements in glucose metabolism and insulin sensitivity as well as adipose tissue dysfunction [12,13]. Interestingly, these effects were also observed after treatment with heat-inactivated (pasteurized) *A. muciniphila*, which appeared to have even more pronounced effects than the live bacterium on many parameters—an effect that was attributed to a specific membrane protein of *A. muciniphila* (Amuc_1100) [13]. Since obesity, gut barrier dysfunction, glucose metabolism, insulin resistance, and adipose tissue dysfunction are all considered to contribute to the development and progression of NAFLD/NASH [14,15], we questioned whether treatment with heat-inactivated *A. muciniphila* may also have beneficial effects in obesity-associated NAFLD/NASH development, in a more long-term chronic disease setting (28 weeks).

For this, we used high-fat diet (HFD)-fed Ldlr−/−.Leiden mice, a translational diet-induced mouse model for NAFLD/NASH that exhibits dyslipidemia (hypercholesterolemia and hypertriglyceridemia), as often observed in patients. In response to a high-fat diet (HFD) with a macronutrient composition and cholesterol content that is comparable to human diets [16,17,18], these mice also develop obesity, insulin resistance, adipose tissue dysfunction [18,19], gut permeability [18,20], and NAFLD/NASH [18,19,20,21,22,23,24,25,26]. Besides reflecting histopathological characteristics of human NASH, these mice have also been shown to reflect many molecular processes that underly the pathogenesis of NASH in patients both on the level of gene expression and metabolites characteristic for NASH [22,23,25]. Ldlr−/−.Leiden mice were fed the NASH-inducing HFD supplemented with heat-inactivated *A. muciniphila* (2 × 10^8^ heat-inactivated CFU per mouse per day, as described by Plovier et al. [13]) for 28 weeks, and the effects of the treatment on obesity and associated metabolic dysfunction in the gut (including microbiota composition), adipose tissue, and liver were studied relative to an untreated HFD control.

## 2. Results

### 2.1. Heat-Inactivated A. muciniphila Did Not Affect Development of Obesity or Associated Metabolic Risk Factors

**Figure 1:**
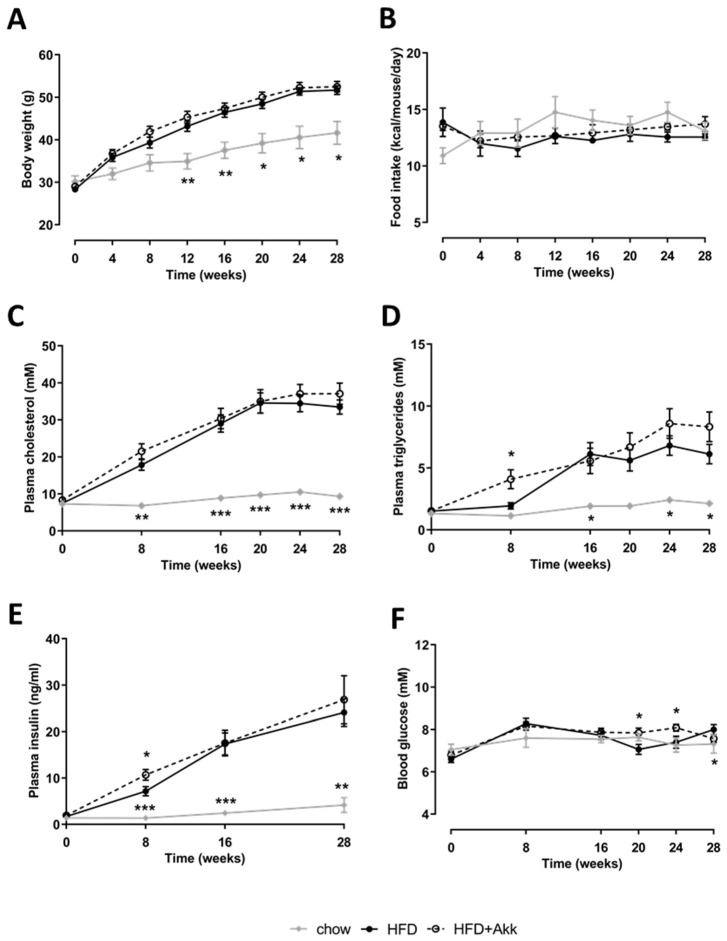
Heat-inactivated *A. muciniphila* did not affect development of obesity or associated metabolic risk factors. Ldlr−/−.Leiden mice were fed a chow reference diet (chow; grey line) or a NASH-inducing high-fat diet without (HFD; black line) or with supplementation of heat-inactivated *A. muciniphila* (HFD + Akk; dashed line) for 28 weeks. (**A**) Body weight; (**B**) food intake; (**C**) fasting plasma cholesterol; (**D**) fasting plasma triglycerides; (**E**) fasting plasma insulin; and (**F**) fasting blood glucose over time. Data shown are mean ± SD. * *p* < 0.05; ** *p* < 0.01; and *** *p* < 0.001 vs. HFD.

HFD feeding significantly induced body weight relative to chow-fed controls, and this body weight gain was not affected by treatment with heat-inactivated *A. muciniphila* (Figure 1A). In line with this, there was no effect of heat-inactivated *A. muciniphila* on food intake (Figure 1B). Similarly, plasma cholesterol and triglycerides were strongly and significantly induced in HFD-fed animals relative to chow, with no effect of heat-inactivated *A. muciniphila* (Figure 1C,D). As is typical for this model, plasma insulin levels rose strongly in response to HFD indicative of insulin resistance, while they increased only slightly over time on chow (Figure 1E). Hyperinsulinemia allowed HFD-treated mice to maintain their fasting blood glucose at levels comparable to chow (Figure 1F). Overall, heat-inactivated *A. muciniphila* had no major effects on blood glucose and hyperinsulinemia except some transient minor fluctuations (Figure 1E,F).

### 2.2. Heat-Inactivated A. muciniphila Had Minor Effects on Gut Microbiota in Ileum and Colon Mucosa and Lowered Circulating SCFA Valeric Acid and Caproic Acid

**Figure 2:**
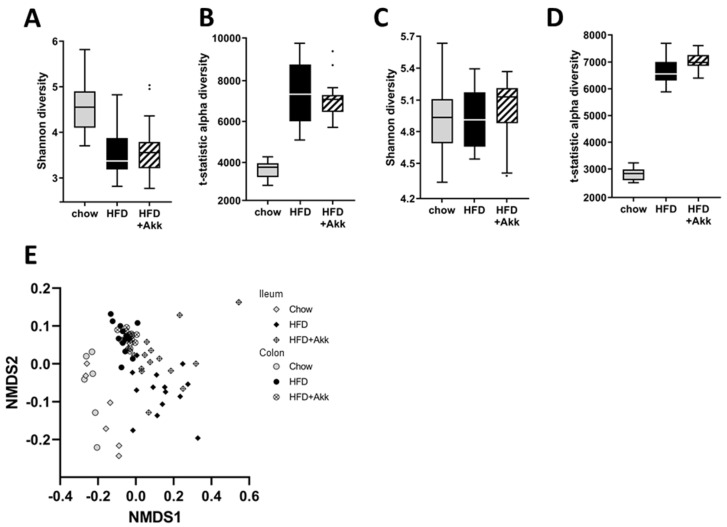
Heat-inactivated *A. muciniphila* has minor effects on mucosal gut microbiota in ileum and colon. Ldlr−/−.Leiden mice were fed a chow reference diet (chow) or a NASH-inducing high-fat diet without (HFD) or with supplementation of heat-inactivated *A. muciniphila* (HFD + Akk) for 28 weeks. (**A**) Shannon index (sensitive for high-abundance bacteria) in ileum; (**B**) tail statistic (t-statistic; sensitive for low-abundance bacteria) in ileum; (**C**) Shannon index in colon; (**D**) tail statistic in colon; (**E**) visualization of total microbiota ordination in ileum and colon with non-metric multidimensional scaling (NMDS) using the Bray–Curtis index.

HFD feeding lowered Shannon diversity, which mainly reflects the diversity of highly abundant bacteria in the ileum mucosa. The tail statistic diversity (mainly reflective of low-abundance bacteria), on the other hand, was increased by HFD feeding in both the ileum and the colon mucosa (Figure 2A,B). Heat-inactivated *A. muciniphila* did not affect microbiota diversity in either of the compartments studied (Figure 2A,B). Analysis of the total microbiota composition showed that the microbiota composition was significantly affected by HFD feeding in both the ileum and the colon mucosa, significantly altering the colonization of numerous genera (Figures S1 and S2). Of note, HFD feeding significantly lowered the abundance of *A. muciniphila* in the colon mucosa (Figure S2). Although the microbiota of the heat-inactivated *A. muciniphila*-treated mice showed great overlap with that of HFD controls (Figure 2E), permutation tests revealed a significant difference between the total microbiota of these two groups in both the ileum (*p* = 0.022) and the colon (*p* = 0.002) mucosa. A more detailed analysis of the genera that were significantly affected showed that the treatment with heat-inactivated *A. muciniphila* resulted in the significantly increased presence of the 16S-rRNA gene of this genera in both compartments, as would be expected after dietary supplementation. In addition, in the ileum mucosa, heat-inactivated *A. muciniphila* lowered the abundance of *Prevotella* and increased the abundance of *Parasutarella* and *Bifidobacterium* (Figure S3). In the colon mucosa, heat-inactivated *A. muciniphila* lowered the abundance of *Corynebacterium*, *Desulfovibrionales*, and *Dorea,* and increased the abundance of *Enterorhabdus* and *Bifidobacterium* (Figure S4).

To investigate potential changes in microbiota functionality, we analyzed plasma levels of short-chain fatty acids (SCFA) as microbiota-produced metabolites (Table 1). This analysis showed that acetic acid, butyric acid, and isobutyric acid were not significantly altered by HFD feeding or by the treatment with heat-inactivated *A. muciniphila*. Valeric acid, caproic acid, methylbutyric acid, and isovaleric acid were significantly increased in HFD animals relative to chow. The HFD-induced increases in valeric acid and caproic acid were attenuated by heat-inactivated *A. muciniphila*, leading to significant reductions for these two SCFA. These results may be indicative of a change in gut microbiota functionality or could point to an altered utilization of these SCFA.

### 2.3. Heat-Inactivated A. muciniphila Lowered Gut Permeability at the End of the Study and Had Minor Effects on Inflammation in Ileum and Colon

**Figure 3:**
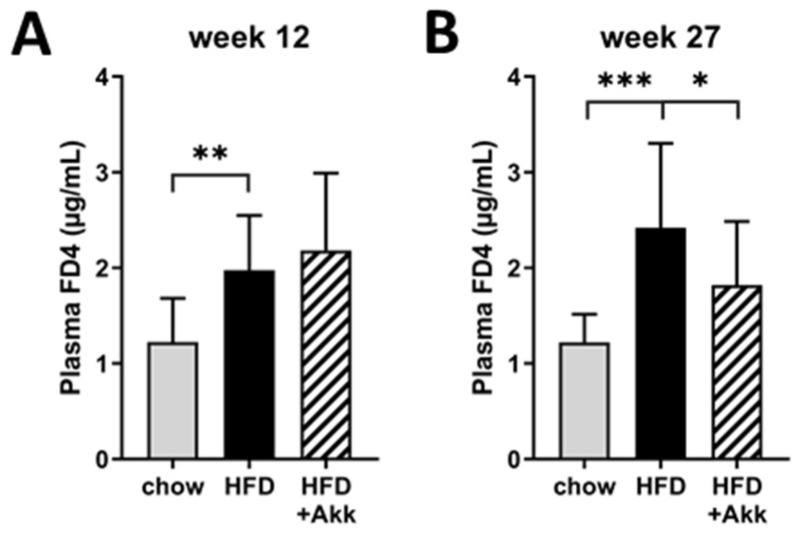
Heat-inactivated *A. muciniphila* lowers gut permeability at the end of the study. Ldlr−/−.Leiden mice were fed a chow reference diet (chow) or a NASH-inducing high-fat diet without (HFD) or with supplementation of heat-inactivated *A. muciniphila* (HFD + Akk) for 28 weeks. Gut permeability assessed by an in vivo functional gut permeability analysis using the Fluorescein isothiocyanate (FITC)-labeled dextran (FD4) assay in (**A**) week 12 and (**B**) week 27 of the study. Data shown are mean ± SD. * *p* < 0.05; ** *p* < 0.01; and *** *p* < 0.001 vs. HFD.

To investigate the effects of heat-inactivated *A. muciniphila* on gut barrier function, we performed an in vivo gut permeability test (FD4 test) in week 12 and 27 of the study. Gut permeability was significantly increased by HFD feeding in week 12 of the study (Figure 3A), particularly at the end of the study (Figure 3B). Heat-inactivated *A. muciniphila* was not able to prevent the initial HFD-induced increase in gut permeability observed in week 12, but prolonged treatment prevented further increase with significantly improved gut barrier function relative to HFD in week 27 (Figure 3A,B).

Next, we analyzed inflammatory mediators (chemokines and cytokines) in ileum and colon tissue to explore tissue inflammation as a potential mediator of the observed effects on gut permeability (Table 2). This analysis showed that HFD feeding did not significantly affect inflammatory chemokine and cytokine content in ileum. Heat-inactivated *A. muciniphila* generally reduced the inflammatory mediators in ileum, non-significantly lowering the protein level of most of the measured factors. A significant reduction was observed in ileal MIP-1a, a chemoattractant for several immune cell types. In colon, HFD feeding generally lowered chemokine and cytokine levels, all non-significantly. Heat-inactivated *A. muciniphila* had mixed effects (mostly non-significant) on inflammatory mediator expression in colon. A significant effect of heat-inactivated *A. muciniphila* was observed on the level of the neutrophil chemoattractant KC in colon, which was increased relative to HFD.

### 2.4. Heat-Inactivated A. muciniphila Did Not Affect Adiposity but Did Improve Adipocyte Hypertrophy in the Mesenteric Depot

**Figure 4:**
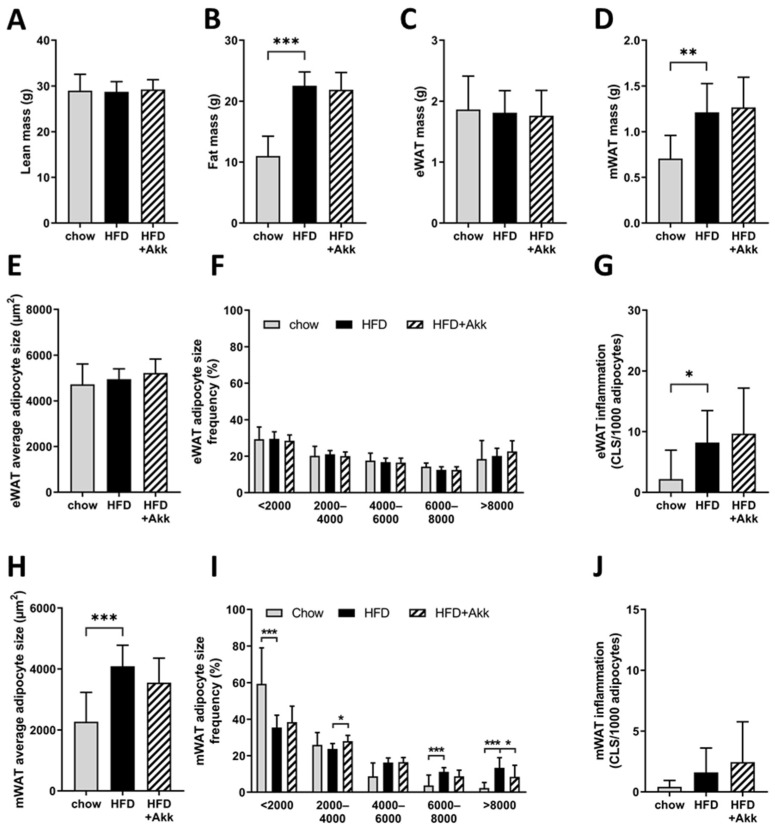
Heat-inactivated *A. muciniphila* did not affect adiposity but did improve adipocyte hypertrophy in the mesenteric depot. Ldlr−/−.Leiden mice were fed a chow reference diet (chow) or a NASH-inducing high-fat diet without (HFD) or with supplementation of heat-inactivated *A. muciniphila* (HFD + Akk) for 28 weeks. (**A**) Total lean mass; (**B**) total fat mass; (**C**) epididymal white adipose tissue (eWAT) mass and (**D**) mesenteric white adipose tissue (mWAT) mass at the end of the study; (**E**) average adipocyte size in eWAT; (**F**) distribution of eWAT adipocytes over 5 size ranges in µm^2^ and (**G**) eWAT inflammation; (**H**) average adipocyte size in mWAT; (**I**) distribution of mWAT adipocytes over 5 size ranges in µm^2^ and (**J**) mWAT inflammation. CLS: crown-like structures. Data shown are mean ± SD. * *p* < 0.05; ** *p* < 0.01; and *** *p* < 0.001 vs. HFD.

Consistent with the observed effects on body weight, HFD feeding significantly increased fat mass (Figure 4A), while lean body mass was not affected (Figure 4B). The epidydimal white adipose tissue (WAT) depot, which is the first depot to expand when body weight increases, was already maximally expanded with both HFD and chow diet, consistent with previous studies [18,26]; however, the weight of the slower responding mesenteric (visceral) WAT depot was significantly higher in the HFD group compared to chow (Figure 4C,D). Heat-inactivated *A. muciniphila* did not alter fat or lean body mass or WAT depot weights (Figure 4A–D). Refined histological analysis of adipose tissue morphometry and inflammation of the epidydimal WAT, however, showed that while average adipocyte size and adipocyte size distribution were comparable in HFD and chow, the number of crown-like structures (CLS; the histological hallmark of adipose tissue inflammation) was significantly increased by HFD feeding (Figure 4E–G). Neither hypertrophy nor inflammation of the epidydimal WAT depot were changed by heat-inactivated *A. muciniphila* (Figure 4F,G).

We next analyzed another visceral fat depot, mesenteric WAT, which is located in close proximity to the intestine. This analysis showed that HFD feeding significantly increased average adipocyte size (Figure 4H), which could be explained by a shift towards a reduction in the presence of small adipocytes (<2000 µm^2^) and an increase in the presence of large adipocytes (6000–8000 µm^2^ and >8000 µm^2^) (Figure 4I). HFD feeding did not yet significantly induce inflammation in mesenteric WAT (Figure 4J). However, heat-inactivated *A. muciniphila* was partly able to prevent HFD-induced hypertrophy in this depot with a tendency (*p* = 0.07) towards reduced average adipocyte size (Figure 4H), a significant increase in the presence of smaller adipocytes (2000–4000 µm^2^), and a significant reduction in the presence of large adipocytes (>8000 µm^2^) (Figure 4I). The number of CLS was not affected by heat-inactivated *A. muciniphila* (Figure 4J).

### 2.5. Heat-Inactivated A. muciniphila Does Not Affect Development of Non-Alcoholic Steatohepatitis or Hepatic Fibrosis

**Figure 5:**
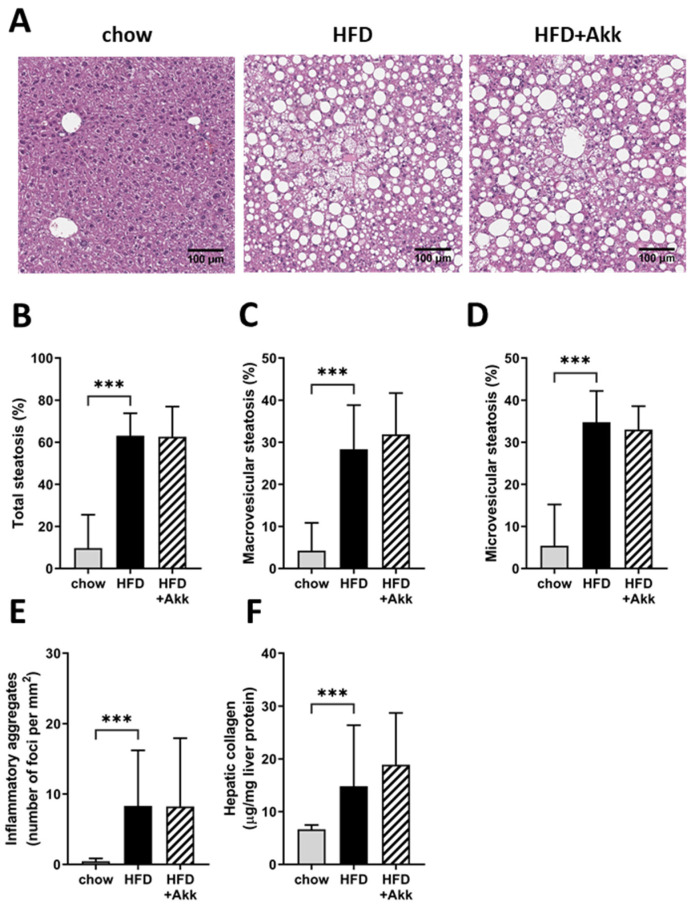
Heat-inactivated *A. muciniphila* does not affect development of non-alcoholic steatohepatitis or hepatic fibrosis. Ldlr−/−.Leiden mice were fed a chow reference diet (chow) or a NASH-inducing high-fat diet without (HFD) or with supplementation of heat-inactivated *A. muciniphila* (HFD + Akk) for 28 weeks. (**A**) Representative photo-micrographs of HE-stained cross sections of the liver; (**B**) total steatosis; (**C**) macrovesicular steatosis; (**D**) microvesicular steatosis; (**E**) hepatic inflammation; (**F**) hepatic collagen content. Data shown are mean ± SD. *** *p* < 0.001 vs. HFD.

Next, we analyzed the development of NASH in HE-stained liver cross sections (representative photomicrographs shown in Figure 5A). HFD-fed controls developed pronounced hepatic steatosis relative to chow (Figure 5B), which was roughly half in the macrovesicular form (Figure 5C) and half in the microvesicular form (Figure 5D). Treatment with heat-inactivated *A. muciniphila* did not influence the development of steatosis in the liver or the distribution of steatosis over the macrovesicular and the microvesicular form (Figure 5A–D). HFD feeding also induced hepatic inflammation relative to chow, which was not affected by heat-inactivated *A. muciniphila* (Figure 5E). Development of hepatic fibrosis was assessed by the biochemical analysis of hepatic collagen content, which showed significant induction in hepatic collagen in HFD relative to chow (Figure 5F). Heat-inactivated *A. muciniphila* did not affect the deposition of collagen in the liver (Figure 5F). In line with these findings on liver histopathology, HFD feeding increased levels of several circulating bile acids (as gut–liver crosstalk mediators), including those increased in human NASH relative to healthy controls [9], which were not significantly affected by heat-inactivated *A. muciniphila* (Table 3).

### 2.6. Heat-Inactivated A. muciniphila Improves the Expression of Biomarkers Associated with Matrix Remodelling and Gut Permeability

HFD feeding increased the plasma concentration of the liver fibrosis marker inhibitor of matrix metalloproteinase TIMP-1, in line with the absence of an effect on hepatic fibrosis heat-inactivated *A. muciniphila*, had no effect on TIMP-1 in the circulation (Table 4). HFD feeding non-significantly increased the levels of the extracellular matrix (collagen) turnover markers PRO-C3 (n.s.), PRO-C4 (*p* = 0.06), C4M (*p* = 0.10), and C6M (n.s.) (Table 4). The trend increase in the type IV collagen production marker PRO-C4 and its respective breakdown marker C4M is noteworthy because this points towards an enhanced matrix-metalloproteinase-mediated turnover of type IV collagen during HFD feeding. Since type IV collagen is a major component of the basal lamina which is critical for gut integrity and gut barrier function [27,28,29], we next investigated the potential effects of heat-inactivated *A. muciniphila* on the matrix turnover markers. *A. muciniphila* treatment was associated with a non-significant reduction in matrix turnover markers PRO-C3 and C6M, a trend towards a reduction in C4M (*p* = 0.09) and a significant reduction in PRO-C4 (Table 4), indicating suppressed turnover of type IV collagen which may be associated with the observed effects on gut permeability.

## 3. Discussion

This study shows that treatment with heat-inactivated (pasteurized) *A. muciniphila* can prevent worsening of HFD-induced gut permeability and has minor beneficial effects on adipose tissue hypertrophy in the mesenteric white adipose tissue, an adipose tissue depot that is anatomically in close proximity to the gut. The effects of heat-inactivated *A. muciniphila* appear to be limited locally, since it is not able to prevent the development of non-alcoholic steatohepatitis (NASH) in livers of Ldlr−/−.Leiden mice, a translational preclinical model for obesity-associated metabolic diseases including NASH [22,23,24,25,30].

Others have reported various metabolic improvements after treatment with *A. muciniphila*, either live or heat-inactivated (pasteurized), in HFD-fed mice using comparable doses. An attenuating effect of *A. muciniphila* on HFD-induced body weight gain has been reported in three short-term studies (4–5 weeks) in C57BL/6 mice that were fed a HFD with a very high (supraphysiological) fat content (60 kcal%) [12,13,31]. In another slightly longer study (10 weeks), using a HFD with a more translational fat content (45 kcal%; the same as in our study), such an effect on body weight was not observed [32], in line with our findings. It is possible that the observed body-weight-attenuating effects, which are thought to result partly from increased energy loss via the feces [31], are dependent on a very high fat content in the diet and cannot be sustained upon long-term HFD feeding. Similarly, in our study, we did not observe any improvement in glucose homeostasis while aforementioned studies using 60 kcal% fat diets reported effects with *A. muciniphila* [12,13]. These improvements in glucose homeostasis could very well be secondary to the observed weight loss in these studies, as lowering body weight in itself would be expected to improve insulin sensitivity [33]. The reported discrepancies between very short (4–5 weeks) and somewhat longer (10 weeks) HFD feeding studies warrant a more rigorous interrogation of prebiotic and probiotic effects in long-term studies, especially because several time-resolved analyses of obesogenic high-fat diet feeding in mice with a C57BL/6 background have shown that time is required to induce pathways of dysmetabolism and inflammation (in liver and WAT) that are relevant for humans [22,34,35,36,37]. Beneficial short-term effects should therefore be interpreted with caution, unless they are replicated under translational conditions of chronic metabolic overload that mimics the chronic state of overweight and obesity in humans.

In agreement with others that have reported improved gut barrier function upon treatment with *A. muciniphila* (indicated by reduced plasma endotoxemia in mice [12,13,38] as well as humans [39]), we observed a reduction in gut permeability in an established functional gut permeability test (used in mice and humans) that measures the ability of the relatively impermeant FITC-labeled dextran (4 kDA) to cross from the intestinal lumen into the circulation [40,41]. These improvements in gut barrier function appear to be unrelated to an improved microbiota composition upon treatment with *A. muciniphila*, since treatment with either live or heat-inactivated *A. muciniphila* is not reported to have any major effects on gut microbiota composition in rodents [12] or humans [39], in line with observations in our study. In addition, the observed effects on gut permeability are unlikely to be the result of a modulation of tissue inflammation in the gut, since chemokine and cytokine expression was not markedly affected by heat-inactivated *A. muciniphila*. Rather, administration of *A. muciniphila* has been shown to restore mucus layer thickness [12] and tight junction expression [13,38] in the gut. In vitro studies in human colonic cell lines have shown that these effects may be mediated by direct adherence of *A. muciniphila* to intestinal epithelial cells [42] or by *A. muciniphila*-derived extracellular vesicles [43]. Furthermore, modulation of SCFA production and/or utilization, as observed herein, can also affect tight junction expression and gut permeability [44,45]. Our observations that heat-inactivated *A. muciniphila* alters functional biomarkers for type IV collagen turnover provide a rationale for an additional complementary mechanism.

More specifically, the observed changes in serum concentrations of PRO-C4 are in support of beneficial effects of *A. muciniphila* on the integrity of the basal lamina of the basement membrane, potentially initiating a repair response whereby type IV collagen is the predominant form of collagen of the basal lamina, i.e., the extracellular matrix layer secreted by mesenchymal and epithelial cells that is critical for gut integrity, host protection, and gut barrier function [27,28,29]. Circulating PRO-C4 and C4M levels tended to increase in response to HFD feeding which could point to an increase in gut basement membrane turnover during obesity-associated increases in gut permeability. Treatment with heat-inactivated *A. muciniphila* significantly reduced PRO-C4 and tended to reduce C4M concentrations, which may be associated with the observed functional improvement in the gut barrier in the FD4 permeability assay.

Similarly, in line with previously reported beneficial effects of *A. muciniphila* treatment on adipose tissue in mice [12,13], we observed some improvement in adipose tissue hypertrophy in the mesenteric depot after treatment with heat-inactivated *A. muciniphila*. More specifically, we observed a shift towards an increased presence of smaller adipocytes (2000–4000 µm^2^) and a reduction in the frequency of very large hypertrophy cells (>8000 µm^2^). However, these effects were not large enough to result in a significant effect on the average adipocyte size or the presence of crown-like structures (considered the hallmark of adipose tissue inflammation) in this depot. While previous short-term treatment studies reported reduced adipose tissue hypertrophy [13] and inflammation [12] in the context of reduced body weight and adiposity, we show that heat-inactivated *A. muciniphila* specifically improves mesenteric adipose tissue hypertrophy independent of effects on body weight. The specificity of the effect on the mesenteric depot is possibly a consequence of its anatomical location, because mesenteric WAT is in close proximity to the intestine, i.e., the primary site in which effects of heat-inactivated *A. muciniphila* can be expected.

Despite these beneficial effects of heat-inactivated *A. muciniphila* on gut permeability and mesenteric adipocyte hypertrophy—which are both thought to drive development and progression of NAFLD, we did not observe any effect of the treatment on a more distant organ, the liver (unchanged liver steatosis, inflammation, or fibrosis). The effects of live *A. muciniphila* on the liver have been studied in one earlier study, which investigated effects on hepatic steatosis in a short (10-week) HFD feeding study in C57BL/6N mice [32]. *A. muciniphila* was found to reduce hepatic steatosis and the liver damage marker ALT in this study. This discrepancy could be attributable to differences in efficacy between treatment with live *A. muciniphila* and heat-inactivated *A. muciniphila*. Although earlier studies have shown that heat-inactivation of *A. muciniphila* enhanced many of the metabolic effects analyzed in that study [13], it is possible that other beneficial effects do require the live bacterium. Furthermore, a major difference between this study and the current study is that the reported disease induction was very mild and was not quantified. In contrast with our study in which NAFLD/NASH was scored quantitatively, the induction of steatosis was mild, and there was no increase in ALT above the normal range or any induction of inflammation or fibrosis. Several time-resolved studies have demonstrated that disease induction in diet-induced models <15 weeks remains modest and is not representative for the severity typically observed in NASH patients [34,36,46].

Our study is the first to investigate the effects of heat-inactivated *A. muciniphila* on development of NAFLD/NASH using a translational model in a chronic disease setting that mimics the clinically relevant severe disease stages. Altogether, our study shows that heat-inactivated *A. muciniphila* can improve obesity-induced gut permeability and associated basal lamina turnover markers (PRO-C4) and has minor effects on tissues in close proximity to the gut, i.e., mesenteric white adipose tissue hypertrophy. Despite these beneficial effects in gut and adipose tissue, heat-inactivated *A. muciniphila* was not able to attenuate the development of NAFLD/NASH.

## 4. Materials and Methods

### 4.1. Culture and Pasteurization of Akkermansia muciniphila

*A. muciniphila* (ATTC BAA-835) was cultured according to the methods described by Plovier et al. [13]. In short, *A. muciniphila* was grown in a synthetic medium (PYG modified) in which 0.05% mucin was replaced by soy peptone (16 g/L), threonine (4 g/L), and a mix of glucose and N-acetyl glucosamide (25 mM each). Cultures were washed in PBS and then inactivated by pasteurization for 30 min at 70 °C, after which they were lyophilized.

### 4.2. Animal Study

The animal experiment was performed in the AAALAC-accredited animal facility at TNO Metabolic Health Research (Leiden, The Netherlands). Ethical approval for this study was obtained from an independent Animal Welfare Body (IvD TNO; approval number TNO-245). The study was performed in accordance with the rules and regulations set forward by The Netherlands Law on Animal Experiments. Male Ldlr−/−.Leiden mice (age 15–17 weeks) were obtained from the breeding colony at TNO Metabolic Health Research (Leiden, The Netherlands). Animals were group-housed in makrolon cages (3–6 mice/cage) in animal rooms with relative humidity of 50–60%, a temperature of ~21 °C, and a 7 am to 7 pm light cycle with ad libitum access to food and water. Before the start of the study, all animals were kept on a standard rodent maintenance diet (9 kcal% fat, 33 kcal% protein, 58 kcal% carbohydrate; R/M-H, Ssniff Spezialdiäten, Soest, Germany). At the start of the study, mice were matched into 3 experimental groups based on body weight and blood glucose. The first group (chow; *n* = 6) was kept on the standard rodent maintenance diet for the remainder of the study. The second group (HFD; *n* = 15) was fed an established NASH-inducing high-fat diet (45 kcal% fat from lard, 20 kcal% protein, 35 kcal% carbohydrate; D12451, Research diets, New Brunswick, NJ, USA) [18,19,22,25]. The third group (HFD + Akk; *n* = 15) was fed the same HFD, supplemented with the pasteurized and lyophilized Akkermansia at a concentration of 7.14 × 10^10^ heat-inactivated colony-forming units (CFUs) per kg diet to establish a dose of 2 × 10^8^ heat-inactivated CFU per mouse per day at an expected food intake of 2.8 g diet per mouse per day. Body weight and food intake were monitored throughout the study and 5 hour-fasted blood samples for EDTA plasma isolation were collected via the tail vein at set intervals. Body composition (fat mass and lean mass) was analyzed by EchoMRI (EchoMRI-LLC, Houston, TX, USA) in week 27 of the study. Gut permeability was assessed in vivo in week 12 and 27 of the study, using a fluorescein isothiocyanate-labelled dextran 4 kD (FD4) gut permeability assay as described previously [18,20]. After 28 weeks, mice were terminated by gradual-fill CO_2_ asphyxiation, after a 5 hour fast. Blood was collected by cardiac puncture for serum and EDTA plasma isolation. The epidydimal white adipose tissue, the mesenteric white adipose tissue, and the liver were isolated and weighed before being partly drop-fixed in formalin and then embedded in paraffin for histological analyses; and partly snap-frozen in liquid nitrogen and then stored at −80 °C for biochemical analyses. The mucosa of the ileum and colon were scraped and collected for analysis of the mucosal microbiota.

### 4.3. Plasma Biochemistry

Blood glucose was measured during blood sampling with a hand-held glucometer (Freestyle Freedom Light, Abbott Laboratories, Lake Bluff, IL, USA). Plasma insulin was measured by ELISA (Chrystal Chem Inc., Downers Grove, IL, USA). Plasma cholesterol and plasma triglyceride levels were measured in freshly prepared plasma using enzymatic assays (CHOD-PAP and GPO-PAP, respectively; Roche Diagnostics, Almere, The Netherlands). Plasma TIMP-1 was measured in terminal plasma by ELISA (R&D systems, Abingdon, UK). All these assays were performed according to the manufacturers’ instructions. Extracellular matrix turnover markers PRO-C3 and PRO-C4 for type 3 and type 4 collagen formation and C4M and C6M for metalloproteinase-mediated degradation of type 4 and type 6 collagen, respectively [47,48,49,50], were measured in terminal serum using competitive ELISAs (Nordic Bioscience, Herlev, Denmark).

### 4.4. Gut Microbiota Analyses

Mucosal samples were collected from the ileum and colon during termination and were immediately snap-frozen in liquid nitrogen and then stored at −80 °C until further analysis. Genomic DNA was isolated using an AGOWA mag mini kit (DNA Isolation Kit, AGOWA, Berlin, Germany) according to the manufacturer’s instructions. Metagenomic sequencing and data analysis were performed as described previously [20].

### 4.5. Gut Chemokine and Cytokine Analyses

Chemokine and cytokine protein levels were measured in tissue homogenates of the ileum and colon. Tissues were homogenized in a 50 mM Tris-HCl pH 7.4, 150 mM NaCl, 5 mM CaCl_2_, and 1% Triton X-100 lysis buffer with protease inhibitors (cOmplete protease inhibitor cocktail, Merck Life Science NV, Amsterdam, The Netherlands). Simoa^®^ multiplex assays were used for analysis of the chemokine panel MIP-1a, IP-10, and KC, and for the cytokine panel IL-10, IL-17, and TNFα (both measured on an SP-X analysis system, all Quanterix, Billerica, MA, USA). RANTES and MIF were measured using DuoSet ELISAs (R&D systems). Protein concentrations were measured in the same homogenates with the BCA Protein Assay Kit (Thermo Scientific, Waltham, MA, USA) and all chemokines and cytokines were expressed per mg protein.

### 4.6. Adipose Tissue Histology

Cross sections (5 µm) of epidydimal and mesenteric WAT were stained with hematoxylin–phloxine–saffron (HPS) and digitized using a slide scanner (Aperio AT2, Leica Biosystems, Amsterdam, The Netherlands) for histological analysis of hypertrophy and inflammation. Adipose tissue morphometry (average adipocyte size and adipocyte size distribution) and inflammation (number of crown-like structures; CLS per 1000 adipocytes) were analyzed as described previously [18,51].

### 4.7. Liver Histology and Biochemical Analysis of Collagen

NASH was analyzed by a board-certified pathologist in HE-stained cross sections (3 µm) of the medial lobe using an adapted grading method for human NASH [52,53]. Two cross-sections per mouse were analyzed for the degree of steatosis (total, macrovesicular, microvesicular) and hepatocellular hypertrophy, all expressed as a percentage of the liver area affected. Hepatic inflammation was analyzed by counting the number of inflammatory foci per field at a 100× magnification in five non-overlapping fields per specimen, expressed as the number of foci per mm^2^. The hepatic collagen content was determined biochemically by hydroxyproline analysis in acid hydrolysates of the left liver lobe, relative to a collagen standard (Total Collagen Assay; Quickzyme, Leiden, The Netherlands). Collagen was expressed relative to liver protein assessed in the same hydrolysate (Total Protein Assay, Quickzyme).

### 4.8. Determination of Short-Chain Fatty Acids and Bile Acids in Plasma

Plasma short-chain fatty acids (SCFA; acetic acid, propionic acid, butyric acid, caproic acid, isobutyric acid, methylbutyric acid, isovaleric acid, and valeric acid) were analyzed at Triskelion (Utrecht, The Netherlands) by ultra-performance liquid chromatography (Ultimate 3000 UPLC; Thermo Scientific, Waltham, MA, USA) coupled to high-resolution mass spectrometry (HR-MS; Q-Exactive mass spectrometer equipped with an electro-spray ionization probe; Thermo Scientific). Proteins were precipitated with 3M zinc sulfate solution. Deuterated internal standards were added and samples were derivatized with glycidyltrimethylammonium chloride. After derivatization, the SCFAs (injection volume 2 µL) were separated on a Waters Acquity HSS T3 column (150 × 2.1 mm, 1.8 µm; Waters Chromatography, Etten-Leur, The Netherlands) using a mobile phase gradient from 99.5% mobile phase A (0.1% formic acid in MilliQ) to 100% mobile phase B (0.1% formic acid in acetonitrile) in 19 min with a flow rate of 0.4 mL/min. Mass detection was carried out using electrospray ionization in the positive mode (spray voltage 3 kV, scan range *m*/*z* 150–700). Propionic acid was below the lower level of quantitation for most samples and these data are, therefore, not reported.

Plasma bile acids (cholic acid, glycocholic acid, taurocholic acid, deoxycholic acid, tauro-deoxycholic acid, chenodeoxycholic acid, β-muricholic acid, tauro-chenodeoxycholic acid, ursodeoxycholic acid, tauro-ursodeoxycholic acid, and hyodeoxycholic acid) were determined at Triskelion by UPLC coupled to HR-MS (Dionex UltiMate 3000 UPLC system coupled with a Q Exactive mass spectrometer, Thermo Fisher Scientific, Breda, The Netherlands, and an Acquity BEH C18 column, 2.1 mm × 50 mm, 1.7 μm, Waters, Etten-Leur, The Netherlands) at 40 °C as described previously [54,55]. Calibration standards were prepared for individual deuterated bile acids at concentrations of 0.005–40 μM. Samples were prepared as previously described [54]. The ratio of mobile phase A (1 mM ammonium formate in water (pH 4.4) to mobile phase B (acetonitrile:water [95:5 *v*/*v*] containing 1 mM ammonium formate) was varied over 14 min, with a flow rate of 600 μL/min. The injection volume was 3 μL and the autosampler temperature was 20 °C. The mass spectrometer, equipped with a heated electrospray ionization source, was operated in negative mode, and full-scan spectra were recorded. The spray voltage was 3 kV, and capillary and probe heater temperatures were 350 °C and 320 °C, respectively. Nitrogen was used as the sheath and auxiliary gas, set at 60 and 20 (arbitrary units), respectively. The resolution was 17,500 at *m*/*z* 250–1000. The data were analyzed using LCQUAN software (Thermo Fisher Scientific, Inc., Waltham, MA, USA).

## Figures and Tables

**Table 1:** Plasma levels of short-chain fatty acids.

	Chow	HFD	HFD + Akk
**Acetic acid (µg/mL)**	2.87 ± 0.68	2.73 ± 0.82	2.54 ± 1.00
**Butyric acid (µg/mL)**	0.04 ± 0.02	0.05 ± 0.02	0.05 ± 0.03
**Valeric acid (µg/mL)**	0.006 ± 0.003 **	0.013 ± 0.005	0.008 ± 0.005 *
**Caproic acid (µg/mL)**	0.10 ± 0.02 ***	0.31 ± 0.13	0.17 ± 0.04 **
**Isobutyric acid (µg/mL)**	0.03 ± 0.01	0.04 ± 0.02	0.04 ± 0.01
**Methylbutyric acid (µg/mL)**	0.03 ± 0.01 *	0.05 ± 0.01	0.04 ± 0.01
**Isovaleric acid (µg/mL)**	0.01 ± 0.00 *	0.03 ± 0.01	0.03 ± 0.01

Plasma short-chain fatty acids were measured in terminal plasma (*t* = 28 weeks). Data shown are mean ± SD. * *p* < 0.05; ** *p* < 0.01 and *** *p* < 0.001 vs. HFD.

**Table 2:** Protein levels of chemokines and cytokines in ileum and colon tissue.

	Chow	HFD	HFD + Akk
**Ileum**			
MIP-1a (pg/mg protein)	6.99 ± 2.16	11.11 ± 4.22	6.80 ± 3.52 *
IP-10 (pg/mg protein)	3.95 ± 1.09	2.93 ± 2.06	1.73 ± 1.40
KC (pg/mg protein)	6.83 ± 2.46	10.54 ± 3.66	9.25 ± 6.08
RANTES (ng/mg protein)	0.66 ± 0.64	0.52 ± 0.38	0.38 ± 0.16
MIF (ng/mg protein)	82.12 ± 42.45	74.93 ± 52.89	68.45 ± 55.98
IL-10 (pg/mg protein)	3.81 ± 2.25	1.90 ± 2.08	1.35 ± 1.37
IL-17 (pg/mg protein)	0.47 ± 0.27	0.88 ± 0.74	0.67 ± 0.63
TNF-α (pg/mg protein)	2.72 ± 1.31	1.84 ± 1.30	1.64 ± 1.26
**Colon**			
MIP-1a (pg/mg protein)	8.64 ± 9.49	4.48 ± 1.19	3.90 ± 0.55
IP-10 (pg/mg protein)	4.80 ± 3.92	2.50 ± 0.75	2.66 ± 0.95
KC (pg/mg protein)	7.28 ± 1.93	7.94 ± 0.81	10.90 ± 3.14 **
RANTES (ng/mg protein)	0.71 ± 1.08	0.20 ± 0.06	0.20 ± 0.04
MIF (ng/mg protein)	84.53 ± 33.06	59.89 ± 39.90	74.63 ± 29.04
IL-10 (pg/mg protein)	4.50 ± 0.46	4.72 ± 2.65	3.44 ± 1.16
IL-17 (pg/mg protein)	1.18 ± 1.03	0.55 ± 0.53	0.43 ± 0.41
TNF-α (pg/mg protein)	3.81 ± 0.94	3.69 ± 1.36	4.22 ± 1.03

Data shown are mean ± SD. * *p* < 0.05 and ** *p* < 0.01 vs. HFD.

**Table 3:** Plasma bile acids are not affected by heat-inactivated *A. muciniphila*.

		Chow	HFD	HFD + Akk
**Classical pathway**				
**Primary bile acids**				
	Cholic acid (µM)	0.39 ± 0.36	0.25 ± 0.17	0.36 ± 0.43
	Glycocholic acid (nM)	2.67 ± 1.36 *	9.92 ± 10.77	13.67 ± 11.98
	Taurocholic acid (µM)	0.10 ± 0.05 **	1.59 ± 1.82	2.06 ± 1.81
**Secondary bile acids**				
	Deoxycholic acid (µM)	0.29 ± 0.22 *	0.53 ± 0.23	0.73 ± 0.39
	Taurodeoxycholic acid (µM)	0.03 ± 0.01 ***	0.24 ± 0.19	0.24 ± 0.13
**Alternative pathway**				
**Primary bile acids**				
	Chenodeoxycholic acid (nM)	6.00 ± 6.48	12.92 ± 9.45	17.27 ± 17.81
	Taurochenodeoxycholic acid (nM)	4.40 ± 2.61 **	77.08 ± 61.25	96.33 ± 73.97
	β-Muricholic acid (µM)	0.36 ± 0.21 *	0.14 ± 0.08	0.25 ± 0.23
**Secondary bile acids**				
	Ursodeoxycholic acid (nM)	30.20 ± 15.99	35.23 ± 20.77	46.93 ± 33.16
	Tauro-ursodeoxycholic acid (nM)	14.50 ± 9.71 **	97.77 ± 68.33	123.40 ± 79.61
	Hyodeoxycholic acid (nM)	13.17 ± 13.23	20.64 ± 9.45	24.40 ± 17.71

Plasma bile acids were measured in terminal plasma (*t* = 28 weeks). Bile acids that are underlined are increased in NASH patients relative to healthy controls [9]. Data shown are mean ± SD. * *p* < 0.05; ** *p* < 0.01; and *** *p* < 0.001 vs. HFD.

**Table 4:** Serum concentrations of extracellular matrix turnover biomarkers.

	Chow	HFD	HFD + Akk
TIMP-1 (ng/mL)	2.03 ± 0.43 **	4.45 ± 1.01	4.71 ± 1.97
PRO-C3 (ng/mL)	11.7 ± 3.77	16.24 ± 7.02	13.86 ± 6.02
PRO-C4 (ng/mL)	132.0 ± 21.27	182.0 ± 58.84	141.3 ± 38.64 *
C4M (ng/mL)	4.23 ± 0.99	5.59 ± 1.59	4.53 ± 1.26
C6M (ng/mL)	4.04 ± 1.45	7.19 ± 3.29	5.89 ± 2.65

Plasma extracellular matrix turnover markers were measured in terminal serum (t = 28 weeks). Data shown are mean ± SD. * *p* < 0.05 and ** *p* < 0.01 vs. HFD.

## Data Availability

The data presented in this study are available in this article and the accompanying Supplementary Material.

## Supplementary Materials

The following are available online at https://www.mdpi.com/article/10.3390/ijms23042325/s1.
