# Supplementary material for: Heat-Inactivated Akkermansia muciniphila Improves Gut Permeability but Does Not Prevent Development of Non-Alcoholic Steatohepatitis in Diet-Induced Obese Ldlr−/−.Leiden Mice
Source: Int J Mol Sci. 2022 Feb 19;23(4):2325. doi: 10.3390/ijms23042325 (PMC8878538; doi:10.3390/ijms23042325)
Supplement: Supplementary file 1 [file ijms-23-02325-s001.zip › ijms-1535647-supplementary.pdf]

International Journal of
Molecular Sciences

MDPI

Supplementary Material

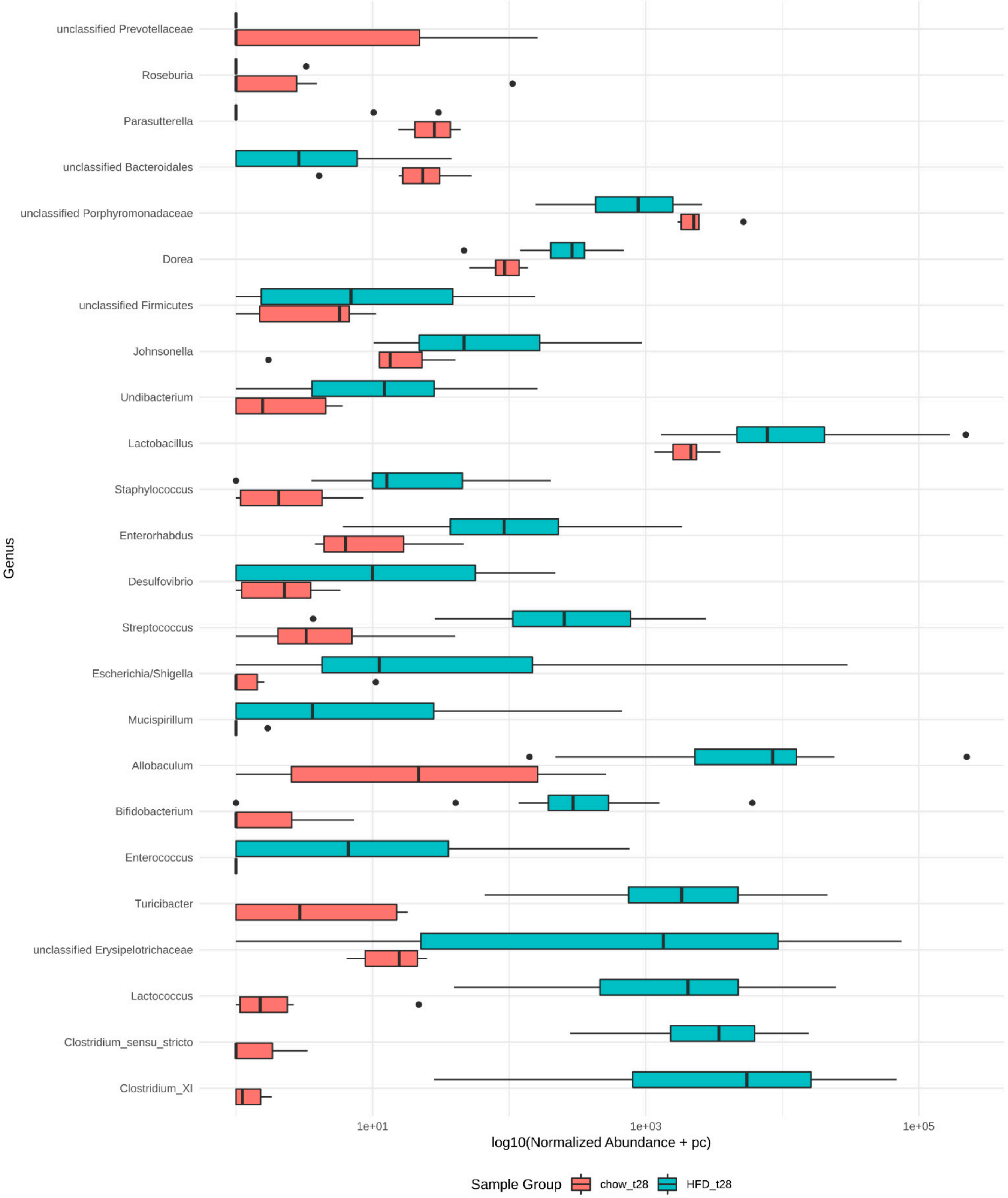

Figure S1. Significantly altered genera in ileum mucosa in HFD relative to chow-fed mice. Ldlr−/−.Leiden mice were fed a chow reference diet (Chow) or a NASH-inducing high-fat diet for 28 weeks. Graph shows the abundance of genera that were significantly altered by HFD feeding relative to chow.

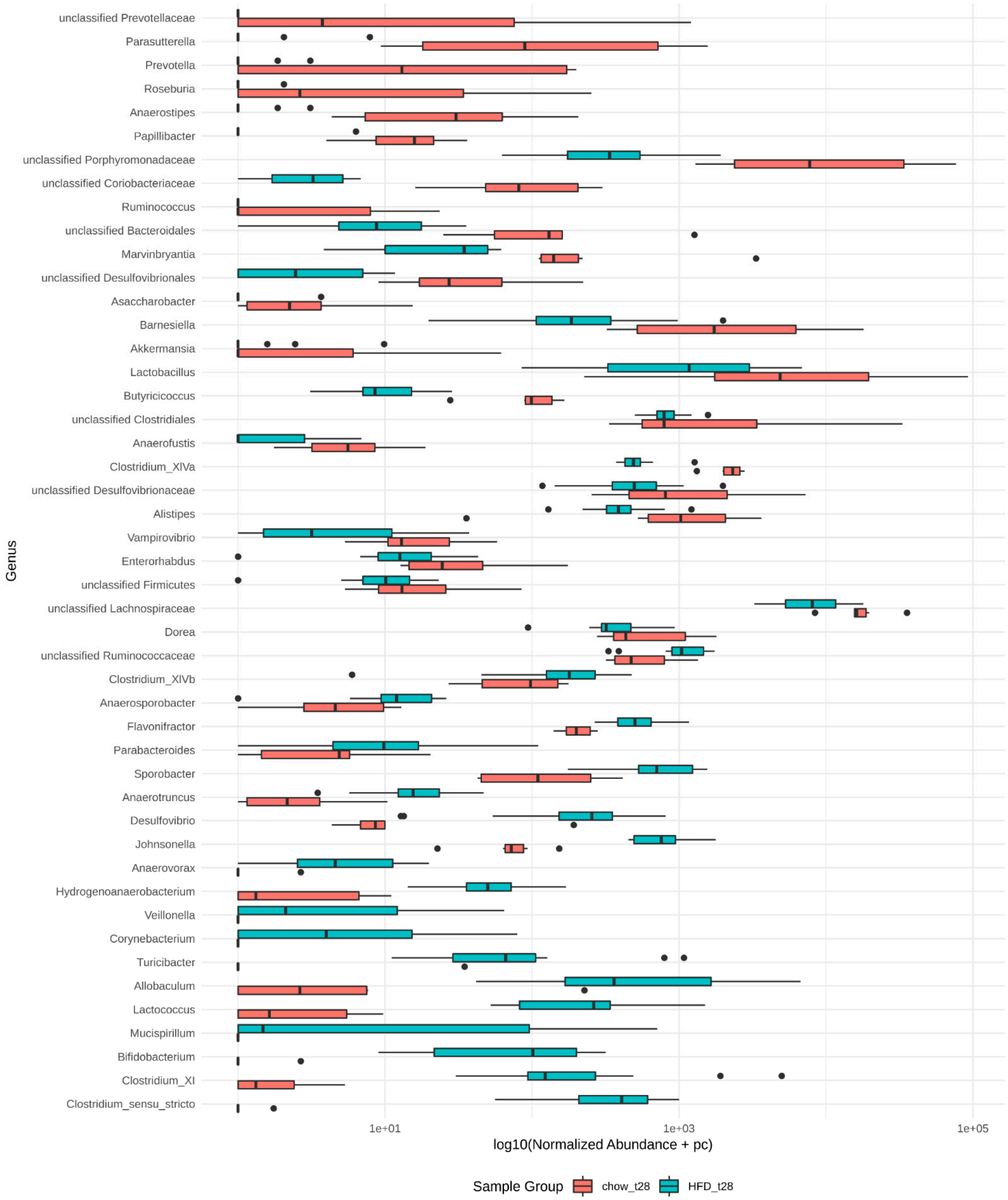

Figure S2. Significantly altered genera in colon mucosa in HFD relative to chow-fed mice. Ldlr−/−.Leiden mice were fed a chow reference diet (Chow) or a NASH-inducing high-fat diet for 28 weeks. Graph shows the abundance of genera that were significantly altered by HFD feeding relative to chow.

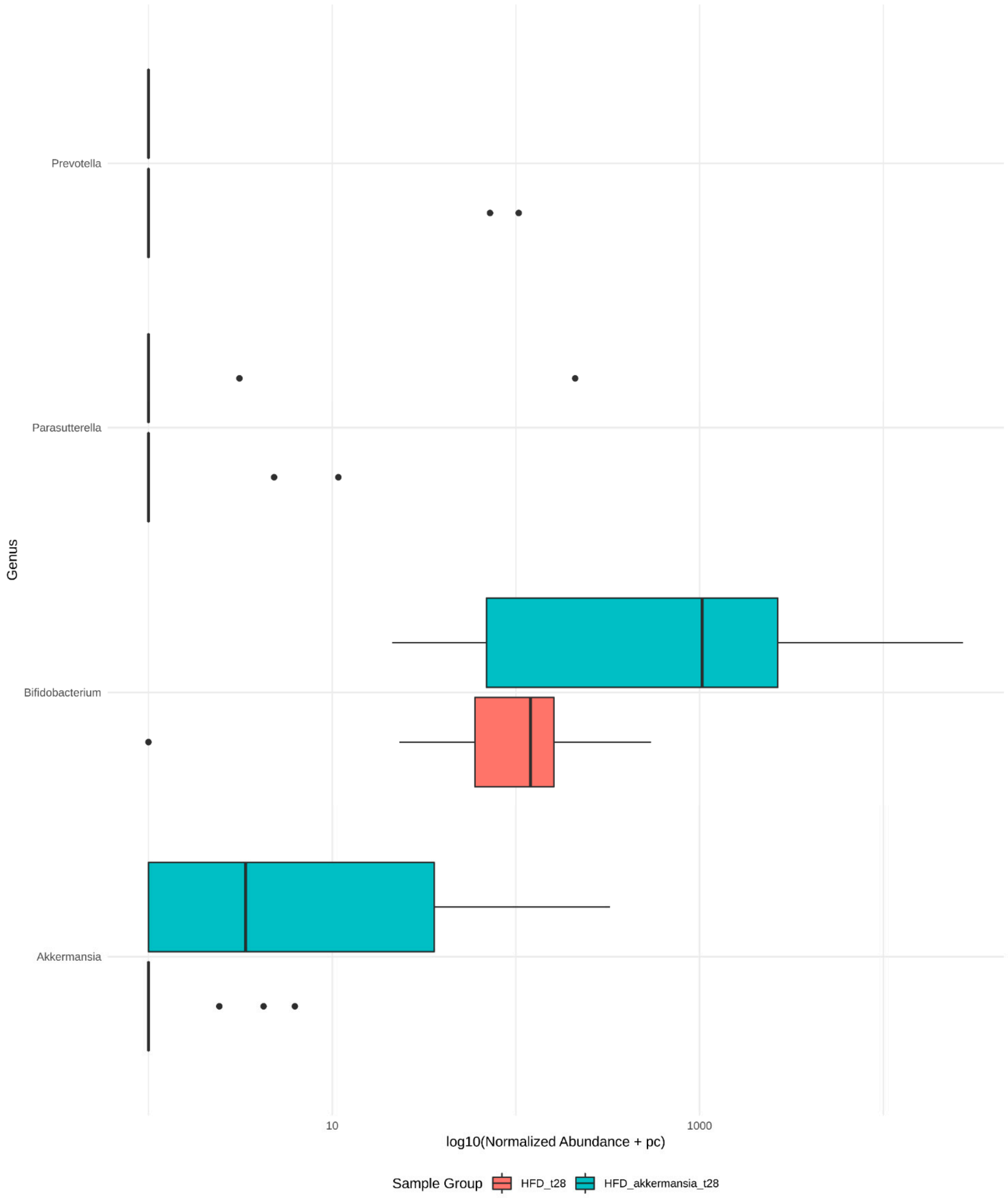

Figure S3. Significantly altered genera in ileum mucosa in HFD + Akk vs. HFD. Ldlr-/-.Leiden mice were fed a NASH-inducing high-fat diet without (HFD) or with supplementation of heat-inactivated A. muciniphila (HFD + Akk) for 28 weeks. Graph shows the abundance of genera that were significantly altered by treatment with heat-inactivated *A. muciniphila* relative to HFD controls.

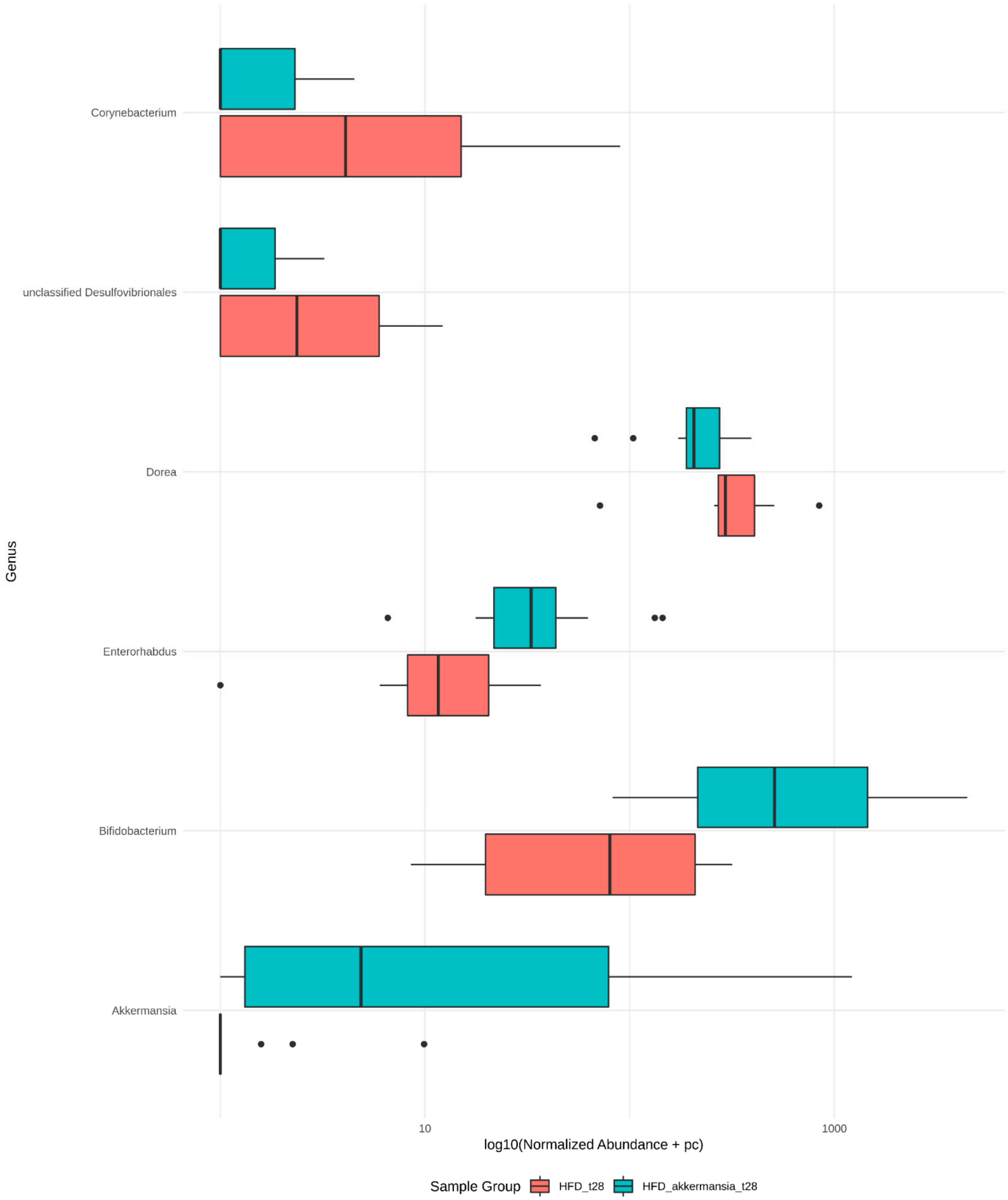

Figure S4. Significantly altered genera in colon mucosa in HFD + Akk vs. HFD. Ldlr−/−.Leiden mice were fed a NASH-inducing high-fat diet without (HFD) or with supplementation of heat-inactivated A. muciniphila (HFD + Akk) for 28 weeks. Graph shows the abundance of genera that were significantly altered by treatment with heat-inactivated *A. muciniphila* relative to HFD controls.
